# GRU Neural Network Improved Bioimpedance Based Stroke Volume Estimation during Ergometry Stress Test

**DOI:** 10.3390/s22207883

**Published:** 2022-10-17

**Authors:** Mike Urban, Michael Klum, Alexandru-Gabriel Pielmus, Falk Liebrenz, Steffen Mann, Timo Tigges, Reinhold Orglmeister

**Affiliations:** 1 Department of Electronics and Medical Signal Processing, Technische Universität Berlin, Einsteinufer 17, 10587 Berlin, Germany; 2 Department of Research and Development, Osypka Medical GmbH, Albert-Einstein-Straße 3, 12489 Berlin, Germany

**Keywords:** bioimpedance, cardiac output, cardiovascular diseases, ECG, GRU neural network, hemodynamic parameters, impedance cardiography, signal processing, stroke volume, ICG

## Abstract

Cardiovascular diseases (CVDs) are one of the leading members of non-communicable diseases. An early diagnosis is essential for effective treatment, to reduce hospitalization time and health care costs. Nowadays, an exercise stress test on an ergometer is used to identify CVDs. To improve the accuracy of diagnostics, the hemodynamic status and parameters of a person can be investigated. For hemodynamic management, thoracic electrical bioimpedance has recently been used. This technique offers beat-to-beat stroke volume calculation but suffers from an artifact-sensitive signal that makes such measurements difficult during movement. We propose a new method based on a gated recurrent unit (GRU) neural network and the ECG signal to improve the measurement of bioimpedance signals, reduce artifacts and calculate hemodynamic parameters. We conducted a study with 23 subjects. The new approach is compared to ensemble averaging, scaled Fourier linear combiner, adaptive filter, and simple neural networks. The GRU neural network performs better with single artifact events than shallow neural networks (mean error −0.0244, mean square error 0.0181 for normalized stroke volume). The GRU network is superior to other algorithms using time-correlated data for the exercise stress test.

## 1. Introduction

In the western world, the demographic change results in an increasing number of older people. This leads to a rise in treatments of different diseases due to a higher prevalence in this age group. The main parts of non-communicable diseases (NCDs) that lead to healthcare intervention are cardiovascular diseases (CVDs). In 2016, 17.9 million people died because of CVDs, according to the World Health Organization (WHO) [1]. This problem will get worse while more and more countries get developed. The two main CVDs are ischemic heart diseases (heart attack) and cerebrovascular diseases like strokes. Both can have a severe course of the disease that results in permanent restriction and immobility or death in a worst-case scenario. Besides the age, different risk factors can increase the prevalence of CVDs like the use of cigarettes, reduced physical activity, an unhealthy diet, and hypertension, to mention just some of them [2]. Another aspect is the enormous rise in healthcare costs, especially in developed countries. The cost of healthcare can develop into a problem for societies due to limited sustainable financial resources. For example, in 2015, Germany spent 13.7% (EUR 46.4 Billion ) of their total annual costs of all diseases to treat CVDs [3]. This is only the cost for the government and does not include the additional payments for employees and employers. With Germany’s general health insurance, this could lead to the question of which treatment shall be paid and to whom. Currently, this only applies to very expensive cancer treatments but should be addressed at an early stage.

CVDs are more than a single and separated risk as a part of NCDs. CVDs themselves can be a risk factor for COVID-19 (coronavirus disease 2019) that is provoked by the SARS-CoV-2 (Severe acute respiratory syndrome coronavirus 2). Patients over the age of 60 with CVDs, hypertension, diabetes, chronic respiratory diseases, and cancer belong to the vulnerable population, according to the WHO [4].

In this regard, prevention, early diagnosis, and personalized therapies are needed to address the problem of the increasing prevalence of CVDs. Generally, different methods are used to identify CVDs, such as the long-term electrocardiogram (ECG), exercise stress test on an ergometer, echocardiogram, coronary angiography, x-ray imaging, radionuclide imaging, as well as magnetic resonance imaging (MRI) and computer tomography (CT). The mentioned methods have different disadvantages, e.g., some are expensive, invasive, and most of them cannot be used in everyday life for a long-term tracing of the patient and its cardiovascular system status.

Ergometry is predominantly used for a first examination and diagnosis because it is non-invasive, inexpensive, and requires a manageable setup and spatial space. During an ergometer stress test, the patient is requested to cycle with an increasing load until the maximum possible physical activity is reached. The ECG is recorded the whole time and evaluated after the stress test. Different morphological changes can be assessed, like an ST-segment depression that can indicate myocardial ischemia, e.g., due to atherosclerosis [5]. Moreover, a 6 min walk test is used to identify cardiovascular problems and evaluate the cardiovascular state of a human, e.g., after heart surgery.

Another method to track the cardiovascular and hemodynamic status of a patient is the thoracic electrical bioimpedance (TEB) measurement that can be used for impedance cardiography (ICG). TEB and ICG are characterized similarly to ergometry as these methods are non-invasive, cost-efficient, and do not need a complex setup. The ICG approach uses a small alternating current that is inserted into a patient with two disposable electrodes. Two additional electrodes are used to measure the resulting surface potential field. This configuration is called a tetrapolar measurement and reduces the error due to the resistance of the measurement wires as well as the electrode to subject interface resistance, of which the main contributor is the stratum corneum of the human skin. A complex bioimpedance signal is calculated with the alternating current and measured voltage. For the following work, the absolute value of the bioimpedance is used for the processing.

### 1.1. Scope and Purpose of the Presented Work

The presented work will improve the data acquisition for ICG signals and stroke volume (SV) determination during strong movements resulting in severe disturbances. The robust measurement can be used for enhanced CVD diagnostics. Therefore, a feasible electrode setup for bioimpedance measurements during an *ergometry stress test* that is also used for a push/pull force stress test is presented. Moreover, the work will introduce the used electronic components and their arrangement and point out the protocol of the automated tests. This work addresses the methodological aspects of SV estimation by an artificial neuronal network with gated recurrent units (GRUs) that transforms an ECG into a bioimpedance signal. It estimates the electromechanical transfer function (the ECG shows the electrical excitation, and the ICG is the electrically measured mechanical response) and can be used to estimate the bioimpedance signal during disturbances on the basis of the far more robust ECG signal. In this work, the proposed GRU network approach is compared to currently used, new, advanced, and adapted signal processing approaches. The suitability for signal denoising is evaluated. It is planned to investigate the clinical implications of this work in the future.

Robust measurement of SV and the bioimpedance signal during ergometry could facilitate the evaluation of bioimpedance signals and derived medical parameters for the diagnosis of possible CVDs, additionally to a generally used ECG recording. An improvement in measurement robustness could also help collect data for long-term measurements in everyday life situations. The general use within an intensive care unit (ICU) or during a general hospitalization would be enhanced by reducing movement artifacts.

The paper is organized as follows. The *state of the art* is depicted, followed by section *materials and methods*, explaining the performed human study and algorithms used. The work ends with the presentation of the *results*, a *discussion* of the findings, and a short *conclusion*. A brief introduction to the ICG fundamentals can be found in the Supplementary Materials.

### 1.2. State of the Art

The most difficult challenge with using bioimpedance measurement during ergometry is the signal processing stage, which must reduce the disturbances due to movement artifacts without or with only minimal reduction or alternation of information of the signal of interest. For filtering purposes, different approaches exist.

Generally, interference removal is performed with high and low pass filters to remove high-frequency disturbances and baseline wander. This simple approach is only possible if the frequency components of disturbances and those of the signal of interest differ by a certain bandwidth. Generally, this is not the case for movement artifacts during bioimpedance measurements. The middle part of the shown signals is disturbed by movement artifacts.

Another approach is calculating the mean of the estimated SV from the disturbed bioimpedance signal for a certain number of beats. The disadvantage of this approach is that the beat-to-beat resolution is missing, and morphological analysis of the bioimpedance signal is challenging. Besides this, many algorithms for fiducial point detection will not work on heavily disturbed bioimpedance signals.

An early, well-working approach is the ensemble averaging (EA) algorithm [6]. EA uses a couple of matching, consecutive signal parts, e.g., between R-R waves. These signal parts get aligned and summed up before they are divided by the number of intervals. This reduces the uncorrelated disturbance of the cardiovascular signal depending on the ensemble size. The approach works well but also reduces the resolution from beat-to-beat to the ensemble size. It produces an average signal on which morphological signal analyses can be performed, and also fiducial point detection is possible. It works best with a relatively stable bioimpedance signal. Therefore, it is not suited for *ergometry stress test* data because the heart rate and morphology of signals will change significantly. Additionally, the approach is blurring the fiducial point of the bioimpedance signal in the time and amplitude domain which can affect point detection algorithms in the post-processing stage.

A more advanced approach is an adaptive filter that uses a noise reference to denoise a signal. The difficulty is in providing this noise reference in clinical practice. This could be done with active electrodes and accelerometers on every electrode due to push and pull forces on electrodes being a major reason for disturbances. With our setup, we also measured the acceleration of every electrode in three dimensions to apply such a filter. An advanced adaptive filter that was recently investigated is the scaled Fourier linear combiner (SFLC) [7]. This filter generates the bioimpedance signal, e.g., the $\frac{dZ\left(t\right)}{dt}$ signal, by combining the fundamental frequency with a certain number of harmonics. The filter coefficients contain the amplitudes of the corresponding harmonics. The fundamental frequency is the reciprocal value of the R-R interval extracted from the ECG. Therefore, it is not a constant frequency. This approach is currently used with different adaptations where systolic and diastolic phases are processed separately [8].

Wavelet transform is another researched approach for denoising of bioimpedance signals [9]. This method works well for baseline wandering as well as high-frequency interference. Movement artifacts that have a frequency spectrum similar to the bioimpedance signal are less reducible due to the approach’s inherent limitation of distinguishing between signal components in the same frequency spectrum.

Other possible approaches that are not currently investigated but could be promising are the empirical mode decomposition (EMD) [10], the principal component analysis (PCA) [11], and the independent component analysis (ICA) [12] with two separate measured bioimpedance signals. Some of these methods fail because they are not able to distinguish between artifacts and bioimpedance signals in the same frequency spectrum.

Currently, neural network approaches for signal reconstruction are used by Yamamoto et al. [13]. This group reconstructed an ECG with Doppler sensor data. Neural networks are also used to predict ECG data [14] and to transfer ECG data from a three-lead ECG to a standard 12-lead ECG (see [15,16]). GRU networks are evaluated to perform sequencing modeling [17]. We combine some ideas for the bioimpedance signal reconstruction during an ergometry test.

## 2. Materials and Methods

### 2.1. Proposed Methods

#### 2.1.1. Shallow Neural Network—(SNN)

**Figure 1:**
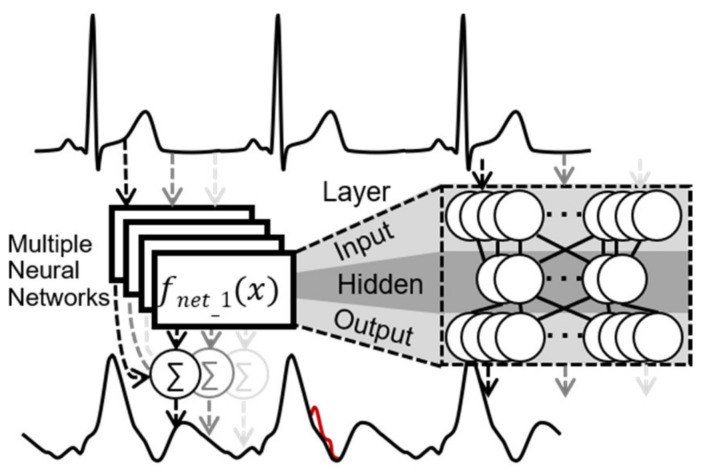
Principle of shallow neural network algorithm—the upper signal is the ECG (input), the lower signal is the bioimpedance signal dZ/dt (output).

An ensemble of 50 simple shallow neural networks is used to estimate the heartbeat temporal aligned transfer function from the ECG to the bioimpedance signal dZ/dt. Each shallow neural network has 200 input neurons and 200 output neurons. The hidden layer is fully connected with 5 neurons. Every single heartbeat is extracted with the support of the Pan-Tompkins algorithm as an R-wave identifier. A heartbeat for this algorithm is defined as follows: The start index of the current heartbeat is the sample index between (in the middle of) the current and the previous R-wave. The end index of the current heartbeat is the temporal middle of the current and following R-wave. The end index is lowered by one sample to prevent overlapping in the heartbeat extraction. The heartbeats are scaled from their original sample length to 200 samples before processing and vice versa after the processing stage of the neural network with linear resampling. Due to this, the length of the input and output data is constant. The output of the simple parallel processed shallow neural networks is merged by summation and normalized by the number of the networks. Due to different starting conditions for the training process and different initial weights, the learned transfer function will be slightly different for every neural network. The merged output shall be a representation of the common subject-specific ECG to bioimpedance signal transfer function. Generally, this works only well if the heart rate is constant. The principle of the shallow neural network data processing flow can be seen in Figure 1. To protect the shallow neural networks from disturbed signals during the training phase, heartbeat signal segments with large artifacts were excluded by calculating the power of the signal.

#### 2.1.2. Gated Recurrent Unit Network—(GRU Network)

**Figure 2:**
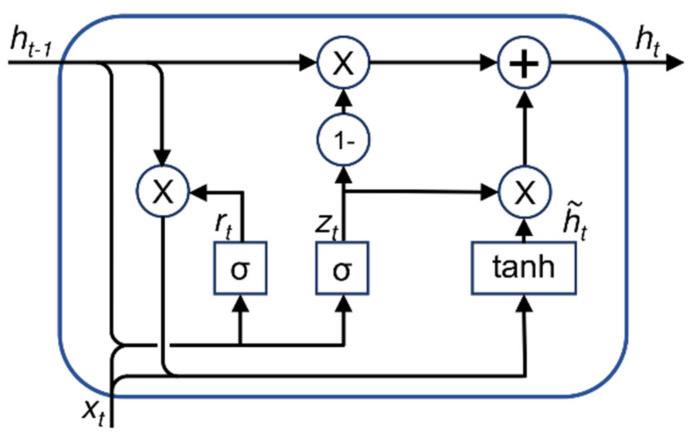
GRU network cell with *h_t_*_−1_ is the hidden state from the last timestamp, *h_t_* is the current hidden state, *x_t_* is the current input, *r_t_* is the reset gate output, *z_t_* is the update gate output, and $\tilde{h}$*_t_* is the candidate hidden state.

The second neural network topology and approach uses gated recurrent units (GRUs). GRUs are used to store, e.g., temporal information without the problem of vanishing or exploding gradients as it is known for general recurrent neural networks. GRU neural networks are a sequential modeling approach compared to shallow neural networks. In contrast to long-short term memory (LSTM) units, GRUs are more efficient with regard to computing time, see Figure 2 for the GRU network cell structure. Compared to our former work with LSTM networks, this is important to reduce the processing time due to the larger dataset [18].

In contrast to traditional shallow neural networks, these types of networks can preserve information for a long time compared to vanilla recurrent neural networks (RNNs). The general idea is an artificial recurrent neural network where the current information is retained for a particular time. With the GRU network, the temporary information of the data is stored depending on its importance for the adaptation of the network to meet the targets. This type of network is used because the heart rate changes significantly during the *ergometry stress test*. This will also change the electro-mechanic transfer function of the heart, meaning that the ECG signal shape and the bioimpedance signal (dZ/dt) will change with the heart rate.

**Figure 3:**
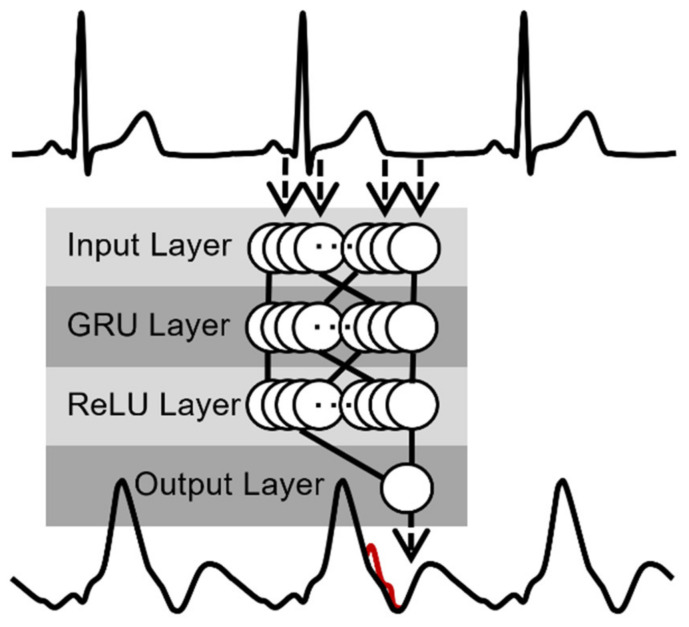
Principle of GRU neural network algorithm—the upper signal is the ECG (input), the lower signal is the bioimpedance signal dZ/dt (output).

The general principle of the GRU network and its data flow can be seen in Figure 3. The input layer consists of 200 neurons, followed by a GRU layer with 200 units, followed by a 200 neuron fully connected rectified linear unit (ReLU) layer, and the network ends in a single neuron. This single neuron represents the current reconstructed or estimated bioimpedance signal sample. This network characteristic is less important for the *force test* with a relatively constant heart rate but more critical for the *ergometry stress test* data. The model’s hyperparameters are optimized with the *force test* data because we have a clear reference. This includes a change in training epochs, the number of GRU units in the hidden layer, and the learning rate. This hyperparameter optimization is performed with 5-fold cross-validation. The best hyperparameter pairs are chosen, and the *force* and *ergometry stress test* data are processed again with the best setting for the later comparison with the algorithms introduced in the following section “Previously Published Methods”. As training data, we used 70% of the signals, 15% for the validation, and 15% for testing. The data for the training, validation, and testing were chosen randomly for every fold. In contrast to the shallow neural network, we do not need any additional information such as R-waves for segmenting the ECG signal. The GRU network input and output work with a sliding window.

#### 2.1.3. Previously Published Methods

##### Ensemble Averaging—(EA)

**Figure 4:**
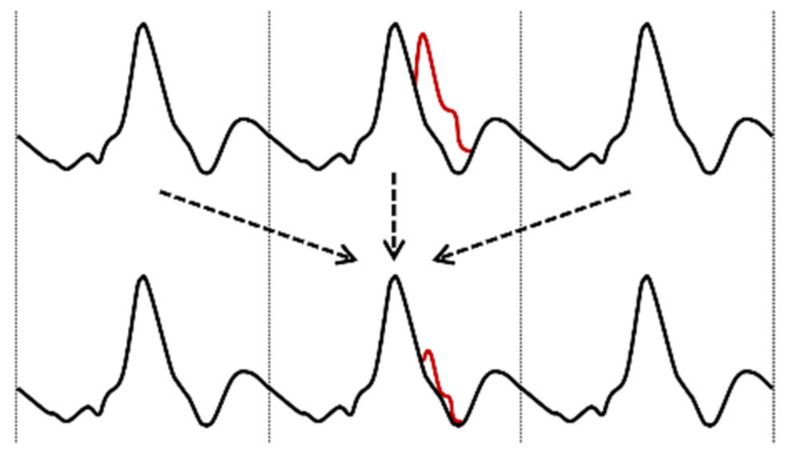
Principle of EA algorithm—black is the bioimpedance signal dZ/dt, red is the artifact in the current heartbeat cycle. Heartbeats around the current beat are aligned, scaled, and merged. The arrows indicate the used heartscycles for the reconstruction.

The ensemble averaging (EA) is one of the first approaches to deal with noise and artifacts. It has been used at least since 1978 [19] and has worked very well with different implementations over the years. This algorithm works as follows: The bioimpedance signal is separated into a certain number of heart cycles. These heart cycles are generally aligned with the ECG signals since the ECG is a more robust signal, and the R-wave is a very prominent and reliably detectable temporal fiducial point. A certain number of bioimpedance heartbeats are then summed and divided by the number of heartbeats (ensembles). This reduces the signal components uncorrelated to the heart cycle by the number of ensembles. A drawback is that the real beat-to-beat resolution is lost, even when for every heart cycle, a bioimpedance signal can be estimated. Common issues are blurring of amplitude and temporal fiducial points, resulting in an averaged signal and a derived averaged SV. For the R-wave detection in our processing of the ECG, the Sedghamiz implementation [20] of the Pan-Tompkins algorithm [21] is used. The ensemble average filtered bioimpedance signal is calculated by

(1) $$\begin{array}{cc} EA\left(i,j\right) & =\frac{1}{N+M+1}\\ & \left(\overset{N}{\underset{n=1}{\sum }}\left(x\left(i-(r_{idx}\left(j\right)-r_{idx}\left(j-n\right))\right)\right)+x\left(i\right)+\overset{M}{\underset{m=1}{\sum }}\left(x\left(i+(r_{idx}\left(j+m\right)-r_{idx}\left(j\right))\right)\right)\right), \end{array}$$

where *x(i)* is the current data point at index *i*, *N* is the number of heartbeats (ensembles) before the current heartbeat (pre-sample), *M* is the number of heartbeats after the current heartbeat (post-sample) and *r_idx_(j)* is the data point index of the *j*-th R-wave corresponding to the heartbeat of the *x(i)* data point. The *j*-th R-wave corresponds to the *x(i)* data point if the index of the data point is between *r_idx_(j)* and *r_idx_(j + 1)*. Depending on the number of ensembles used after the current data point, the signal is processed delayed. The general principle can be seen in Figure 4. The marked red disturbance is reduced by building a temporally averaged signal.

##### Scaled Fourier Linear Combiner—(SFLC)

**Figure 5:**
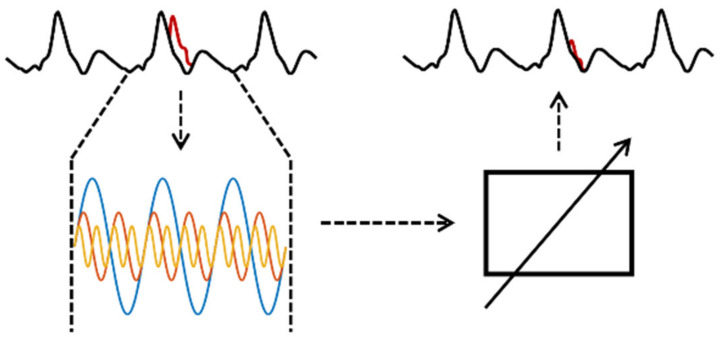
Principle of SFLC algorithm—black is the bioimpedance signal dZ/dt, red is the artifact in the current heartbeat cycle. Base frequency and harmonics depending on R-R-interval are combined by a weighting of frequencies and composition to the estimated signal. The different colors indicate the different harmonics.

The scaled Fourier linear combiner (SFLC) uses previously acquired temporal data information to estimate the current signal. Generally, the SFLC is an adaptive filter that merges a basic frequency, sometimes called the fundamental frequency, and a certain number of harmonics (sine and cosine waves) to an estimated signal of the current heartbeat. The estimated bioimpedance signal is the sum of these frequency components. The weights of the SFLC correspond to the amplitude of the frequency components. The algorithm has been used for bioimpedance signals since 1995 and was established by Barros et al. [7]. This first implementation for bioimpedance signals uses the Widrow-Hoff least mean square (LMS) algorithm for the adaptation of filter coefficients. The estimated signal can be expressed by

(2) $$\begin{array}{cc} SFLC\left(j,i\right) & =\\ & \overset{H}{\underset{l=1}{\sum }}A_{l}\left(j\right)\cdot \cos\left(2\pi \frac{l}{r_{idx}\left(j+1\right)-r_{idx}\left(j\right)}i\right)+\overset{H}{\underset{l=1}{\sum }}B_{l}\left(j\right)\cdot \sin\left(2\pi \frac{l}{r_{idx}\left(j+1\right)-r_{idx}\left(j\right)}i\right), \end{array}$$

where *H* is the number of used harmonics, *A_l_*, and *B_l_* are the filter coefficients of the *l*-th harmonic and the *j*-th R-R-interval, *r_idx_(j)* is the data point index of the *j*-th R-wave, and *i* is the sample index within the current R-R-interval. The SFLC works well as it compares its output with the current signal and adapts its weight, e.g., with the LMS algorithm. After the learning phase, the estimated signal is a representation of the heartbeat correlated signal components of the bioimpedance signal. Like the EA algorithm, the SFLC blurs the signal’s fiducial points in time and amplitude, resulting in an averaged signal containing heartbeat-correlated components with reduced beat-to-beat resolution. The effect of averaging depends on the used adaptation rate for the LMS algorithm needed for updating the weights. With the SFLC, every bioimpedance signal part and beat can be estimated as long as the disturbances do not affect the ECG R-wave detection. The general principle of composing sine and cosine signals with adapted amplitudes and fundamental frequency and the corresponding harmonics can be seen in Figure 5. Ono et al. used the SFLC algorithm to estimate the systolic time intervals during bicycle exercise on impedance cardiography data [22].

##### Adaptive Filter—(AF)

**Figure 6:**
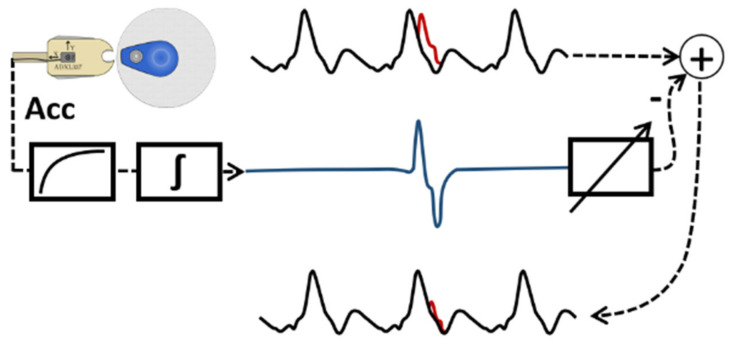
Principle of AF algorithm—black is the bioimpedance signal dZ/dt, red is the artifact in the current heartbeat cycle, blue is the processed acceleration signal.

The general adaptive filter (AF) uses a noise reference signal to remove signal disturbances. As noise references, different sources can be used. The reference has to be uncorrelated to the signal of interest but should be correlated to the noise components of the measured signal. For our approach, we use the acceleration of the electrodes as noise reference since it is commonly known that push-pull forces on electrodes result in movement artifact for the bioimpedance signal. The acceleration signal of the electrodes was preprocessed as follows. First, we used a high pass filter to remove static acceleration due to earth’s gravity. Secondly, we integrated the zero mean acceleration signal twice. The resulting noise reference is strongly correlated with movement artifacts in the dZ/dt bioimpedance signal. Acceleration data for denoising of a biological signal as the photoplethysmogram (PPG) is commonly used, e.g., by Volmer et al. [12]. First, we generated an averaged signal by using both bioimpedance measurements. Second, we generated error signals with every acceleration signal and a separate adaptive filter that estimates the correlated noise components in the acceleration signal and the bioimpedance signal. As the final step, we removed all estimated errors from the bioimpedance signal. The bioimpedance signal is calculated by

(3) $$AF\left(i\right)=x\left(i\right)-\overset{P}{\underset{p=1}{\sum }}\epsilon_{p}\left(i\right),$$

with *x(i)* as the disturbed bioimpedance signal, *P* as the number of acceleration channels (18 due to 6 electrodes with 3 accelerations each) and *ϵ_p_(i)* as the estimated error by an adaptive filter of a single acceleration. Compared to EA and SFLC, the AF algorithm uses additional information from another sensor to reconstruct the bioimpedance signal. This has different advantages, such as no blurring due to averaging and that the actual beat-to-beat resolution is preserved. The drawback is a higher hardware effort due to additional components and a higher software effort due to many processed signals. The principle of our adaptive filter processing can be seen in Figure 6. Because of the signal processing of each acceleration, this is computationally more intensive than the EA and SFLC algorithm.

#### 2.1.4. Hyperparameter Optimization

For all mentioned algorithms, we performed an empirical hyperparameter optimization. As a result, we use 2 pre-samples and 2 post-samples for the EA algorithm. The SFLC uses 10 harmonics and an adaptation rate of 0.05. The shallow neural network uses 50 parallel networks and 5 neurons in the hidden layer. The maximum epochs for the GRU network were 20, the minibatch size was 20, the gradient threshold was 1, and the number of k-folds was 5.

#### 2.1.5. Stroke Volume Estimation

Before and after the signal processing stage, the SV is calculated by determining the fiducial points (B-, C-, and X-point) in temporal matter and amplitude. For the *ergometry stress test*, the SV before the processing stage is only calculated for the baseline and recovery phase, as well as for the breaks with no intentional motion artifacts. Outliers beyond two times standard deviation are excluded. The remaining SV data points are used to generate an SV trend line with modified Akima piecewise cubic Hermite interpolation, see [23,24], to prevent excessive over- and undershooting of the trend line.

#### 2.1.6. Subject Population and Meta Data

We performed the study with 32 human subjects approved by the ethics committee of the Technische Universität Berlin, Department of Psychology and Ergonomics under the tracking number “Urban 01-2018” following the Declaration of Helsinki. Additionally, due to SARS-CoV-2, measurements done since the year 2020 are performed with rigorous hygiene and healthcare precautions approved by the leader of the presidential office of the Technische Universität Berlin. The used medical data were anonymized and therefore are not subject to the EU-DSGVO, according to EU-DSGVO recital number 26. Table 1 depicts the meta-data of the performed human subject study. The body composition was measured with Omron scale BF511. We used 23 datasets. 9 datasets were excluded for this work since they need a complex pre-processing due to large non-physiological single artifacts that may be produced by EM interference and also low ECG signal quality due to dried electrode contact gel.

### 2.2. Data Acquisition Hardware

**Figure 7:**
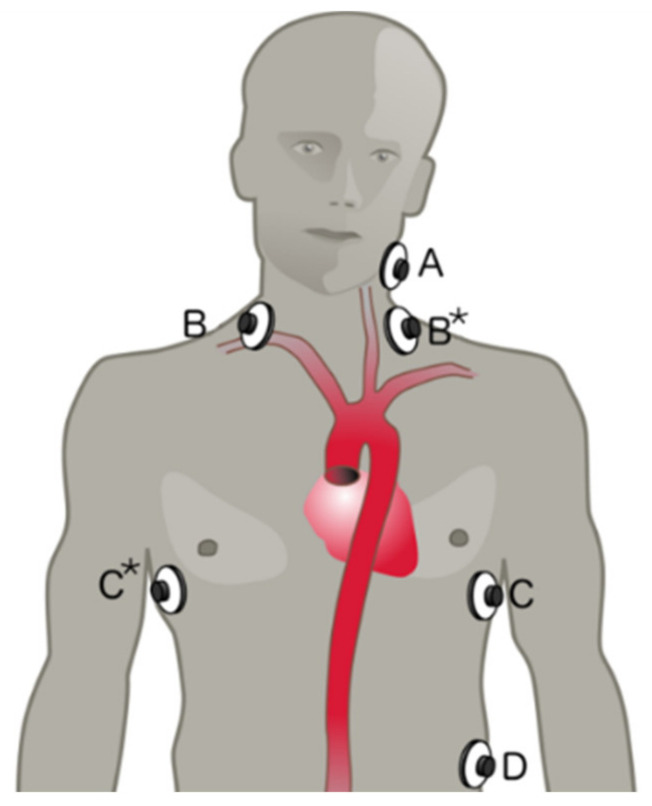
Front view of a subject with electrode positions—electrode A and D inject the current, electrode B/B* and C/C* are used to measure the surface potential.

**Figure 8:**
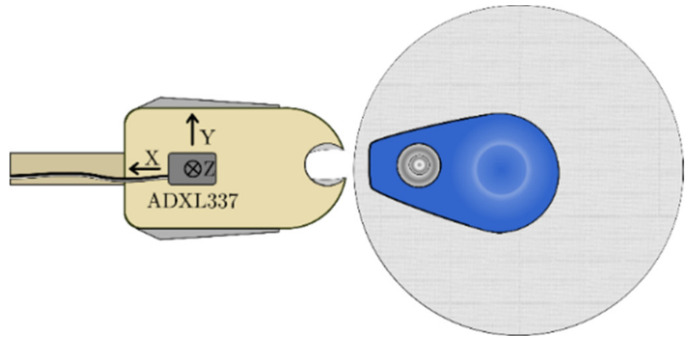
Disposable electrode and clip with the accelerometer.

To perform two synchronous measurements of the bioimpedance of a human subject, we use the medical-grade bioimpedance monitor ICON CORE™ from Osypka Medical GmbH (Berlin, Germany). This device generates a 50 kHz 2 mA_RMS_ alternating current with constant amplitude injected by two disposable electrodes and measures the bioimpedance with two additional electrodes (tetrapolar measurement, electrodes A, B, C, and D; see Figure 7). Additionally, we developed a second measurement board based on the AD637ARZ (RMS-to-DC converter) that uses the ICON CORE™ injected current, and measures the surface voltage drop between separately placed disposable electrodes (B* and C*, see Figure 7). The printed circuit board with the AD637ARZ has a differential pre-amplifier, an optional band-pass for noise reduction, and a separate output driver. As electrodes, we used Ambu^®^ BlueSensor VL electrodes (Ambu GmbH, Bad Nauheim, Germany) due to the very low resistivity of 650 Ω @10 Hz and even lower resistivity at 50 kHz (measured acc. to ANSI/AAMI EC12:2000/(R)2005) with a large adhesive surface for improved fixation even during the *ergometry stress test* and with strongly transpired subjects. Furthermore, we measured the acceleration of every electrode separately with the 3-axis accelerometer ADXL337 (see Figure 8) (Analog Devices, Inc., Wilmington, USA) bandwidth-limited with class 1 ceramic capacitors to 50 Hz. The analog output signals of the ADXL337 were digitalized with the National Instruments™ data acquisition device NI-DAQ USB-6363 (National Instruments, Austin, USA) with a sampling rate of 1000 Hz. The acceleration data were filtered and downsampled to 200 Hz. 200 Hz is the base sample rate for all following processing steps and signals and is also used by the ICON CORE™ (Osypka Medical GmbH, Berlin, Germany) and the second bioimpedance measurement. The systems are synchronized using a trigger signal, captured by all independent measurement units. A general workstation PC is used for data acquisition and study control. The data processing is executed with a high-performance PC. The neural network approaches were calculated with an RTX 3080 graphics card from Nvidia (Nvidia Corporation, Santa Clara, CA, USA), and all other algorithms were performed with an AMD Ryzen 5900 CPU (Advanced Micro Devices, Inc., Santa Clara, CA, USA) to shorten the processing time. In addition, we also acquired the bioimpedance of the right forearm and a photoplethysmogram (PPG) of the right index finger with a device developed by our institute, as well as SV and blood pressure data with the Finapres^®^ NOVA device (DEMCON Focal, Enschede, The Netherlands).

### 2.3. Data Acquisition and Processing Software

For data acquisition, various software is used. First of all, we used iControl™ (Osypka Medical GmbH, Berlin, Germany) to acquire the bioimpedance data, the ECG data from the ICON CORE™ device, and also to collect the data from the AD637ARZ (called CCM—cardiometry current meter) (Analog Devices, Inc., Wilmington, NC, USA) that performs the second bioimpedance measurement. The NI-DAQ (National Instruments Data Acquisition Device) USB-6363 device data were acquired with a non-blocking MATLAB^®^ implementation due to MATLAB^®^ (The MathWorks, Inc., Natick, MA, USA) is also used to control the automatic guided human study. Furthermore, the signal processing and evaluation are implemented to work with the parallel computing toolbox from MATLAB^®^. We measured the second acquired bioimpedance signal with both systems to recognize possible temporal drift within the independent measurement systems ICON CORE™ and NI-DAQ USB-6363. This also helps to track possible missing data blocks. The recorded drift within the measurement time was below one data sample and could be ignored.

### 2.4. Study Protocol and Hygiene Concept

All human subjects were introduced to the study protocol before signing informed consent forms and then participating in the study. An anamnesis was conducted by one study operator to exclude human subjects with potential cardiovascular diseases and to collect the meta-data.

The electrodes for the ICON CORE™ and AD637ARZ were placed as given in Figure 7.

**Figure 9:**
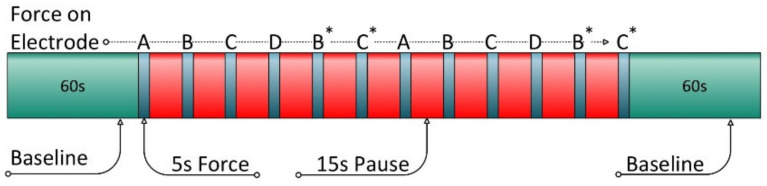
Timeline *force test*—green-marked are baseline at the start and the end, red-marked are the pauses between the disturbances and the letter indicates the corresponding electrode with push/pull forces. The asterix marks the electrodes for the second parallel measurement.

The human subject study was split into two different parts. First, a *force test* part was performed. In the *force test*, a push/pull force is applied to all electrodes separately. This is done twice per subject after a baseline phase and followed by a resting phase. All instructions to apply the push/pull forces or to indicate the baseline and resting phases were controlled by MATLAB^®^ and displayed on a monitor. This was done to achieve a highly reproducible and controlled study environment with a minimum of human interaction with the subject under study. See Figure 9 for the *force test* protocol. With the *force test*, the number and setting of the hyperparameters of the algorithms are evaluated. This is possible because disturbances are only present on a single voltage measuring electrode. The second simultaneous measurement records an undisturbed reference signal.

**Figure 10:**
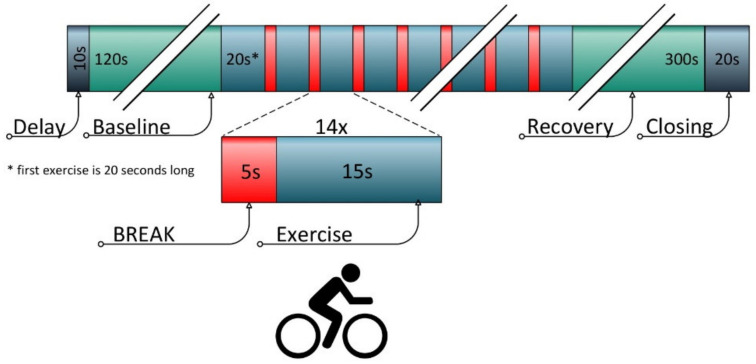
Timeline *ergometry stress test*—green-marked are baseline phase at the start and recovery phase at the end, red-marked are the breaks between the exercise parts.

As the second part of the human subject study, an *ergometry stress test* was performed. The *ergometry stress test* also starts with a resting period to measure the baseline of all signals. This is followed by cycling interrupted by breaks to have reference periods to determine the SV during the *ergometry stress test*. The test is finished with a recovery phase (see Figure 10).

As a cycle ergometer, we used the Christopeit AL 1 (Christopeit-Sport GmbH, Velbert, Germany). The subjects were instructed to have a cadence of 57 rpm at the highest pedal resistance of stage 8, resulting in 20 km/h (equivalent). That should ensure that the movement artifacts and the heart rate during exercise differ with respect to their frequency. It will improve the ability to differentiate between movement artifacts and heart activity, notably if the heart rate rises during the test execution. That is valid for heart rates different from multiples of the cadence.

**Figure 11:**
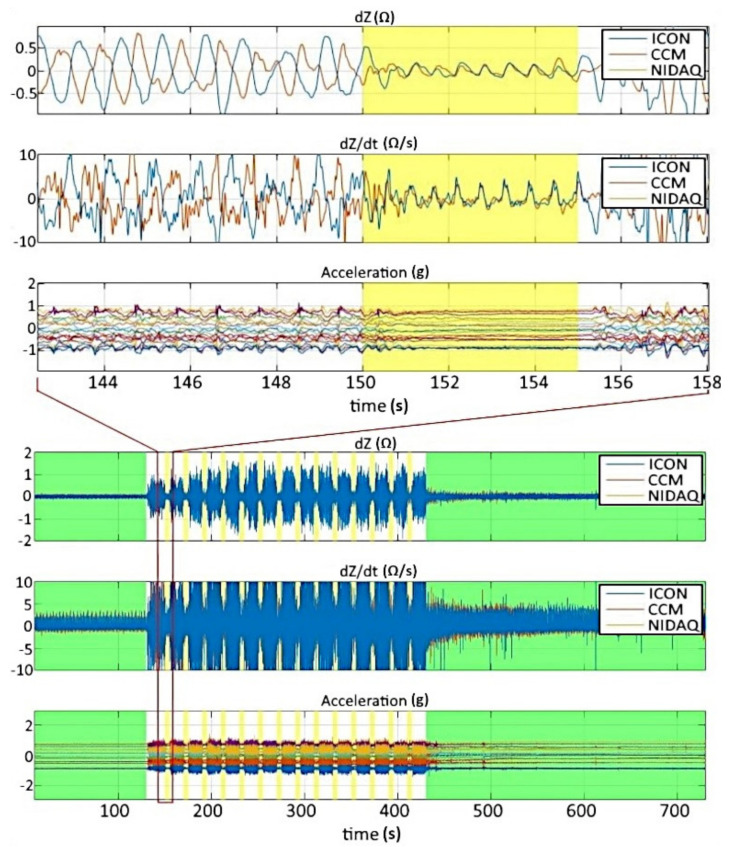
Raw data from the *ergometry stress test* after the acquisition—the lower part shows complete signal (dZ, dZ/dt, and acceleration), the upper part shows zoomed version—baseline and recovery phase are marked green, breaks during cycling are marked yellow (subject 6). Disturbances in both bioimpedance signals are inversely phased and diminish with a delay after the start of the break.

The raw data were inspected and evaluated after the acquisition with MATLAB^®^. Figure 11 shows an example of a part of the signals and data acquired during the *ergometry stress test* of subject number 6.

The bioimpedance signals dZ and dZ/dt, as well as the data from the acceleration, are shown with clearly disturbed signal parts. The dZ and dZ/dt signals acquired by the NIDAQ USB-6363 are congruent with the signals acquired by the ICON CORE™ via the CCM. As formerly mentioned, this shows the synchronization between the different pieces of equipment (ICON CORE™ and NIDAQ). The first green marked area is the baseline phase, and the green marked area at the end is the recovery phase. Within these phases, no significant artifacts occur. The middle part represents the cycling interrupted by breaks. The breaks are marked yellow. The zoomed version of the data (marked red) shows that the disturbances within the bioimpedance signals during the breaks are nearly gone. Because of this, we can calculate valid hemodynamic parameters in these sections. Later on, these calculated values of SV are used to create an interpolation between the breaks. The temporal unit is second (s), and the units for the dZ and dZ/dt signal are Ohm (Ω) respective Ohm per second (Ω/s). The unit of the acceleration is (g), with 1 g being earth gravity.

### 2.5. Spectral Analysis

**Figure 12:**
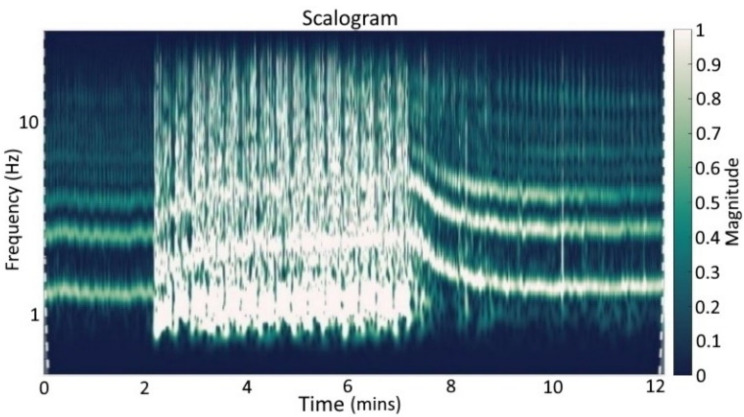
Scalograms of continuous 1-D wavelet transform (Morse wavelet) of dZ/dt signal during *ergometry stress test* before signal processing (subject 6).

We used the scalograms of the continuous 1-D wavelet transform to analyze the acquired signal, see Figure 12. As one can see, the baseline phase and the recovery phase are free of disturbances. The fundamental frequency and the harmonic frequencies rise during the exercise part and fall with the beginning of the recovery time. This was expected and agreed well with the study protocol shown in the figures before. The fundamental frequency is related to the heart rate (e.g., 60 bpm corresponds to 1 Hz) with harmonics multiple times the fundamental frequency. It was expected that harmonics occur since only sine and cosine have a single frequency component, with all other periodical signals consisting of harmonics. Within the exercise part, times with no disturbance as intended are present. Furthermore, the spectrum of the movement artifacts strongly overlaps with the spectrum of the bioimpedance signal, and a general-purpose high or low pass filter will not be able to distinguish between the disturbance and the signal of interest, see Figure 12.

### 2.6. Reference Signal

For the *force test*, we generated a reference signal with the bioimpedance data acquired from both separate and synchronous measurements (from electrode B to C and B* to C*, respectively). That could be done because both separate measurements contain nearly the same signal of interest but uncorrelated disturbances. The reference was generated first by calculating the mean of both separate measurements. This reduces any disturbance—also those that occurred without intended push/pull forces—by a factor of two. Additionally, we replaced the intentionally disturbed signal parts with the undisturbed and isochronously acquired ones from the parallel measurement, e.g., if push/pull forces were applied to electrode B or C, the reference signal uses the measurement from electrode B* and C* and vice versa. The most significant disturbances are related to the voltage measuring electrodes (B, C, B*, and C*). These disturbances can be canceled out with this approach.

The approach is not suitable for the *ergometry stress test* because we have no undisturbed signal parts for every point in time. Therefore, we included breaks to have undisturbed phases. We can calculate all hemodynamic parameters within these phases with a changed cardiovascular status due to the exercise stress test load. In the formerly published article [18], we used these breaks to evaluate our algorithms. This time we generate an estimated signal, to be precise, an estimated SV trend for the whole execution time with the breaks used as support points. After the signal processing with the different algorithms, the SV is calculated, and the result of the SV based on processed data is compared with the estimated trend based on support points. A beat-to-beat reference during the ergometry cannot be reached with any known non-invasive approach and healthy human subjects so far. Besides the ergometry that is a dynamic exercise load, it is also possible to generate a static exercise load. With this static exercise, also called isometric exercise, e.g., wall sit or low squat, it could be easier to measure the bioimpedance. Since a static load did not result in the same cardiovascular response as a dynamic load, and ergometry is used to identify CVDs, we decided to use our approach with a dynamic load and a cycle ergometer [25].

### 2.7. Performance Metrics

As a performance metric, we calculate the signal to noise ratio (SNR) for the *force test* data. This is possible since the *force test* data has a measurement reference because only one voltage deriving electrode is disturbed at one time. The SNR is calculated by

(4) $$\mathrm{SNR}=10\;\cdot \log_{10}\left(\frac{\sum x{\left(i\right)}_{ideal}^{2}}{\sum {\left(x{\left(i\right)}_{ideal}-x{\left(i\right)}_{processed}\right)}^{2}}\right),$$

where the *x(i)_ideal_* is calculated by the average of the two independently measured bioimpedance signals with motion artifacts replaced by the undisturbed measurement channel. *x(i)_processed_* is the bioimpedance signal after the artifact removal. For the *ergometry stress test*, we skipped this metric since we did not have a noise-free reference for this part of the study. We also calculated the mean error (ME), the mean square error (MSE), and the normalized mean square error (NMSE) by

(5) $$\mathrm{ME}=\frac{1}{N}\;\cdot \overset{N}{\underset{j=1}{\sum }}\left(SV{\left(j\right)}_{processed}-SV{\left(j\right)}_{ideal}\right)$$

and,

(6) $$\mathrm{MSE}=\frac{1}{N}\;\cdot \overset{N}{\underset{j=1}{\sum }}{\left(SV{\left(j\right)}_{processed}-SV{\left(j\right)}_{ideal}\right)}^{2}$$

for both studies, with the exception that during the exercise parts *SV(j)_ideal_* is calculated from the SV trend line. The NMSE is calculated by

(7) $$\mathrm{NMSE}=\overset{N}{\underset{j=1}{\sum }}\frac{{\left(SV{\left(j\right)}_{processed}-SV{\left(j\right)}_{ideal}\right)}^{2}}{SV{\left(j\right)}_{ideal}^{2}}\;\cdot 100\%.$$

**Figure 13:**
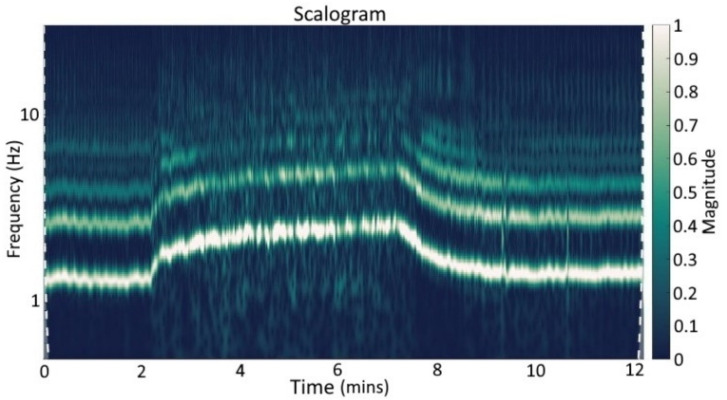
Scalograms of continuous 1-D wavelet transform (Morse wavelet) of dZ/dt signal during *ergometry stress test* after signal processing with GRU neural network (subject 6).

As a frequency analysis approach, we use the scalogram of the continuous 1-D wavelet transform. This is used to present the frequency representation before (see Figure 12) and after the signal processing (see Figure 13).

## 3. Results

For the *force test* with indented push and pull forces on the disposable electrodes, the SV is relatively constant. The intended artifacts caused by push and pull forces can lead to higher or lower SV values, as can be seen from the disturbed signal within Table 2. The SV was normalized to the baseline phase for a better comparison between subjects. Because of that, the calculated parameters are without a unit. The SNN outperforms the other algorithms in mean deviation, mean error (ME), mean square error (MSE), and normalized mean square error (NMSE) regarding stroke volume. The standard deviation of the stroke volume based on the GRU neural network processed signal is close to the reference signal. All algorithms can reduce the intended introduced artifacts and lower the error metrics, even the EA and SFLC algorithms that tend to blur the artifacts and could lead to slightly different calculated stroke volume values close to the temporal occurrence of the disturbance.

We also calculated the performance metrics for the signal waveform; see Table 3 for details. The GRU neural network is superior to the other algorithms for the SNR and the MSE calculated between the processed and the reference signal. The SNN is the best according to NMSE. All algorithms improved the estimation of the signal waveform. The adaptive filter performs the worse for all waveform-related metrics in the *force test*.

For the *force test,* we can conclude that all algorithms can perform a reduction of disturbances for intended introduced artifacts by push and pull forces. Concerning the SNR and MSE, the GRU NN is superior. The best SV estimation was based on the SNN. Despite this, we recommend using the GRU-based approach due to the better representation of variety in SV, e.g., related to respiration. If only an average representation of an ICG signal is needed, the SNN would be the best approach.

The *ergometry stress test* results shown in Table 4 depict the error metrics for the calculated SV from the reconstructed bioimpedance signal in relation to the estimated trend line generated by the baseline and recovery phase and also with the breaks as support points. The EA algorithm is superior for the MSE and the NMSE regarding the normalized stroke volume. The ME of the SV calculated on the SFLC algorithm processed signals is the best for the *ergometry stress test*. The stroke volume calculated by the neural network processed signals (SNN and GRU NN) is underestimated, whereas EA and AF algorithms slightly overestimate the SV. The absolute mean error of the AF algorithm is lower than for the SNN and GRU NN approach, but the MSE is higher. That indicates that SV estimation of AF processed signals is more volatile.

Figure 13 shows the scalograms of the continuous 1-D wavelet transform of the dZ/dt signal for subject 6 after the processing with the GRU network as an example for the *ergometry stress test*. The basic heart rate and its harmonics can be seen clearly. Furthermore, the reconstruction seems to get worse with increased harmonics frequency, and the frequency components are hidden in the noise. The most significant information and signal energy are located in the basic heart rate and lower harmonics. The heart rate variation based on respiration is preserved within the baseline phase. Within the recovery phase, singular unintended artifacts are extenuated. During the exercise, slight heart rate declines can be seen, representing the intended breaks. These declines were not visible before due to the surrounding disturbances.

**Figure 14:**
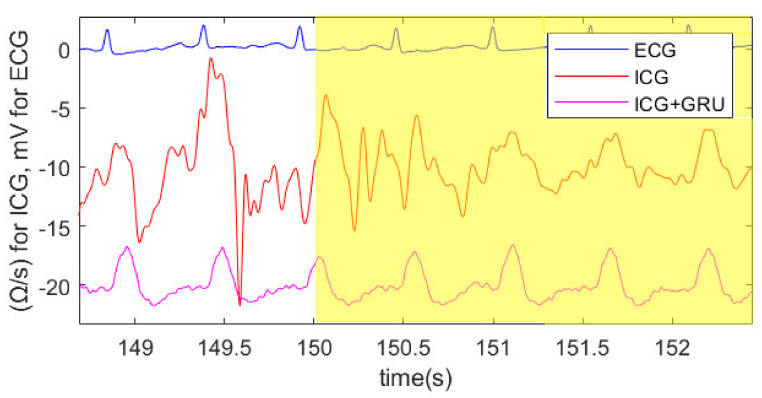
ECG, ICG, and processed ICG with GRU at the transition from the exercise part to the first break (marked yellow) for subject 6. ICG and the processed ICG signal have an offset of −10/−20 Ohm per second (Ω/s), respectively.

Figure 14 shows the result of the GRU network ICG processed signal at the border of the first cycling part to the first break. The intended disturbances slightly vanish after the stop of the cycling. The unprocessed ICG signal needs some heart cycles to stabilize. The GRU-processed ICG signal offers the possibility of fiducial point detection for stroke volume estimation within the formerly disturbed signal part. The ECG signal is more robust than the ICG signal; therefore, heartbeat-correlated signal processing approaches such as EA, SFLC, and SNN can be used. R waves of the ECG signal are clearly visible.

## 4. Discussion

As can be seen in Table 2 and Table 3, the multiple SNNs and the GRU network-based approach perform well and are superior to other algorithms for the *force test*. The mean value of the SV is close to the undisturbed signal for both approaches (1.01 for the SNN and 0.99 for the GRU network). The ME value of the GRU network is negative (−0.0244) and indicates that the SV is estimated to be slightly lower than the reference value based on the undisturbed reference signal. MSE and NMSE show that the SNN is slightly better than the GRU network for this type of test with a relatively constant heart rate. On the other side, the standard deviation of the calculated SV for the SNN (0.056) is lower than the standard deviation of the reference signal (0.070). This can be an indication that the shallow neural network is reproducing a single learned heartbeat for the bioimpedance signal. Generally, heartbeat signals recorded as ECG and also bioimpedance signals differ slightly during the test, especially due to the respiration phases. Because of that variation in R-R interval and ECG heartbeat signal shape, a GRU-based network could be preferred as it could learn the variations of beats by inhalation and exhalation phases. The standard deviation of the GRU network (0.069) is marked in bold because it is closest to the standard deviation of SV of the reference signal (0.070). The SNR (7.34 dB) and the MSE (0.21 (Ω/s)^2^) for the signal waveform of the GRU network are superior to the other algorithms, whereas the NMSE (20.84%) is the best for the SNN approach.

The performance of the multiple SNNs in estimating transfer function based on the ECG signal was assumed to be good because Atoui et al. used this approach to synthesize a 12-lead ECG based on a three-lead ECG [15]. We adapted this idea for the usage of estimation of the ICG signal.

This evaluation was done for the first time with a GRU network and multiple subjects. For further investigation, it seems that this kind of setup is suitable for trials of other different approaches, such as empirical mode decomposition (EMD), principal component analysis (PCA), and independent component analysis (ICA).

For the *ergometry test*, the performance and error metrics are shown in Table 4. The EA and SFLC algorithms perform very well. The SFLC has the lowest ME (0.0) value, whereas the EA algorithm has the lowest MSE (0.045) and NMSE (3.115%). This is the best result for all algorithms. The SFLC reached ME (0.0) due to SV being overestimated for the signal recorded from B* to C* with 0.05 and underestimated for the signal recorded from B to C with 0.05 (SV values normalized).

The first types of algorithms, EA and the SFLC algorithm, use the information within the signal of interest from other timestamps than the current reconstructed signal part. The second type of algorithms, such as AF, SNN, and the GRU NN, use information time-correlated from other sources (acceleration, ECG) to the current reconstruction. Referring to this difference, the GRU is superior for the second type of algorithm, with −0.165 for ME, 0.085 for MSE, and 5.645% for NMSE.

The evaluation of the data, error metrics and reconstructed signal during the *ergometry stress test* is far more complicated. We used the modified Akima piecewise cubic Hermite interpolation to estimate the SV trend line between the breaks of the exercise. This shall give a first estimation of the SV because it is not possible to generate a reference during the cycling parts with a non-invasive approach. It is assumed that the physiological adaptation of the SV during exercise raises and follows a continuous and regular trend as the heart rate does. The heart rate trend could be seen in the recorded data since the ECG is not disturbed as much as the bioimpedance signal.

Figure 13 shows the result of the processing as a scalogram of the continuous 1-D wavelet transform of dZ/dt for the GRU network approach used for subject 6 and the *ergometry test*. As can be seen in comparison with Figure 12, the single fundamental frequency and harmonics of the bioimpedance signal are very well reconstructed. The SFLC algorithm would generate an even clearer frequency analysis, due to the approach’s inherent functionality in estimating the fundamental frequency and harmonic components. For the SFLC algorithm, this type of interpretation based on frequency components is unsuitable.

Figure 14 shows the ECG, ICG, and processed ICG signal by the GRU network. As can be seen, the ICG signal before the break is reconstructed. This figure also shows that the disturbances of the artifacts only subside after a certain delay in the ICG signal.

The algorithms based on information from the ICG signal and other timestamps within it (EA, SFLC) and the algorithms based on correlated data such as ECG and acceleration (AF, SNN, GRU) have each different drawbacks and advantages. The EA and SFLC are more robust in artifact removal but blur the signal, whereas AF, SNN, and GRU preserve the beat-to-beat information. A stacked signal processing system could overcome the different disadvantages. This system could combine the estimation of, e.g., the SFLC and the GRU approach by calculating the energy of the disturbance within a heart cycle and the surrounding disturbances and estimating the ECG-correlated energy level. Depending on the current disturbance and practical scenario, an improved estimation could be given. This could be different from our aim for measuring ICG data and fast changes in SV values in ergometry tests, which lead to a need for less averaged or even beat-to-beat data. For long-term supervision of SV values with only slightly varying signal waveforms and parameters, EA and SFLC may be preferred.

Compared to our former work [18], we could use significantly more subjects, tried to find a first method to evaluate the movement artifacts disturbed signal parts, and improve the investigation of inter-subject phenomena. An advantage of the current GRU-based approach is that it is not necessary to have an ECG QRS-complex detection as is needed for the EA, SFLC, and SNN approach. This reduces the need for additional processing of the ECG signal. The estimation of the ICG signal may be improved by refining the ECG data selection used for the GRU network. For further tests, we also want to investigate heartbeat-aligned data, as it is done for the SNN. The heartbeats are detected for the EA and SFLC anyway. On the other hand, it could also be useful to improve the used signal quality estimator for a better selection of the less disturbed bioimpedance signal parts for the GRU network learning phase. This signal quality estimation was currently done with a calculation of the energy of the signal parts. A third improvement could be made by using more data from all subjects to create a pre-trained network and retrain the network with the training data of the current subject. We have also to improve our evaluation part and may use a static load (isometric exercise) and a dynamic load for the evaluation process.

The currently unused data needs preprocessing, especially for the acceleration signals but also partly for the ECG signal. We had significant artifacts within the acceleration data that could indicate a connection problem at the sensors. Some ECG and bioimpedance data show a large baseline wander within the baseline and the recovery phase that can indicate a bad disposable electrode contact often caused by dried electrodes. The electrode Ag/AgCl gel is necessary for a proper signal and data recording for the ECG and also for the bioimpedance signal.

The MATLAB^®^-based automated guided study protocol was a significant improvement for a reliable and reproducible data acquisition, and we will use this for all following recordings. This also has the advantage that we could minimize the operator and subject interaction between the tests and reduce the risk of a SARS-CoV-2 infection.

We are confident that an improvement of the mentioned aspects could lead to a setup and approach useful for the evaluation of bioimpedance signals and SV determination during *ergometry stress tests*. With this setup and approach, it could be possible to investigate the suitability of bioimpedance measurement as an additional parameter for the diagnosis of CVDs, currently mainly based on ECG signals. The GRU network approach seems to be promising for a further investigation of SV estimation during *ergometry stress tests*.

The average age of the study population is lower than the typical use case (27.17 years). The ergometry stress test is usually performed with middle-aged persons with intermediated probability. Despite this, the new technique must be qualified to work with healthy subjects before it is used with subjects with potential cardiovascular diseases. ST-segment depression that could occur during an ergometry stress test is permanent if the exercise load is maintained or increased. This encourages us that even the EA and SFLC, which perform temporal averaging, as well as the AF, SSN, and GRU approach, will enhance the bioimpedance signal quality and improve CVD detection in elderly subjects.

The needed lower average age for the first test offers the opportunity to investigate the ergometry stress test and the determination of SV for sports cardiology. This could be a potential improvement for performance or recovery determination.

We do not increase the exercise load compared to the generally used ergometry stress test procedure. This was done to ensure a constant cadence that is distinguishable from the heart rate in the first step. Generally, a step-wise increase of exercise load could be performed. The current approach that uses intended breaks, this was simplified as the main aim was to increase heart rate and SV and determine them during significant artifacts.

Further information on the fundamentals of the ICG signal can be found in the Supplementary Materials.

## 5. Conclusions

The aim of the work was to investigate the usage of GRU-based ICG signal estimation and to compare this approach with SNN and commonly used methods.

For the *force test*, we can conclude that single artifacts that occur within the bioimpedance signal can be removed by the GRU network without the disadvantage of blurring the signal, as it is done with the EA and SFLC algorithm. The GRU performs similarly to the SNN approach with a ME of −0.0244, a MSE of 0.0181, and a NMSE of 1.68% (SV error metrics normalized). It underestimates the SV slightly with 0.99 compared to 1.01 for the reference signal (normalized values). As an advantage compared to the SNN, the standard deviation of the GRU is closest to the reference SV values (0.069 for GRU to 0.07 for reference SV), indicating that it learns more than a single ECG to ICG beat representation as heart cycles differ with respiration.

For the *ergometry stress test*, the EA algorithm performs the best for the *ergometry stress test*, with 0.045 for the MSE and 3.115% for the NMSE (SV values are normalized to baseline). The SFLC has the lowest ME for SV values with 0.0. These algorithms use heartbeat time-correlated data before (SFLC) or also after (EA) the current reconstructed heartbeat interval. The AF, SNN, and GRU neural network approach use data from other sources (ECG, acceleration) time-correlated with the current reconstructed signal part. The GRU could outperform the AF and SNN approach but was not as good as the EA and SFLC, with a ME of −0.165, a MSE of 0.085, and a NMSE of 5.645%. Currently, the results show that it is possible to reconstruct a bioimpedance signal disturbed by movement artifacts with a GRU network. GRU is superior in the group of non-blurring algorithms (GRU, AF, and SNN) and is recommended for fast heart rate changes that occur in ergometry.

## Figures and Tables

**Table 1:** Subject meta-data.

Parameter	Number	Percentage	
Subject	23	100%	
Male	16	69.57%	
Female	7	30.43%	
Smoker	4	17.39%	
**Parameter**	**Mean**	**Median**	**Std.**
Age	27.17 year	25 year	+/−6.46 year
Height	1.77 m	1.775 m	+/−0.108 m
Weight	72.42 kg	74.7 kg	+/−17.3 kg
BMI	22.87 kg/m^2^	22.33 kg/m^2^	+/−3.84 kg/m^2^
Sports	2.22 tpw *	3 tpw *	+/−1.13 tpw *
Body Fat	21.52%	19.5%	+/−7.64%
Visceral Fat	5.04%	4%	+/−3.44%
Muscle Mass	36.64%	38.1%	+/−5.78%

* tpw—times per week.

**Table 2:** SV results for force tests.

SV	Mean	STD	ME	MSE	NMSE [%]
Referenze Signal	1.01	0.070			
Disturbed Signal	1.16	0.303	0.1447	0.2052	19.6
EA	1.05	0.107	0.0398	0.0509	4.86
SFLC	1.11	0.178	0.0995	0.0983	9.59
AF	1.06	0.218	0.0484	0.0806	7.71
SNN	**1.01**	0.056	**0.0017**	**0.0162**	**1.51**
GRU NN	0.99	**0.069**	−0.0244	0.0181	1.68

SV deviation calculated to reference signal for the signal processing methods of the *force test*; best values are marked in bold, SV values are normalized.

**Table 3:** ICG signal waveform performance metrics for force tests.

Signal Waveform	SNR [dB]	MSE [(Ω/s)^2^]	NMSE [%]
Disturbed Signal	−0.81		
EA	5.16	0.49	59.11
SFLC	3.32	0.71	81.38
AF	1.98	0.98	107.11
SNN	7.28	0.22	**20.84**
GRU NN	**7.34**	**0.21**	22.17

Performance metrics related to the ICG signal waveform for the signal processing methods of the *force test*; best values are marked in bold.

**Table 4:** SV results for ergometry tests.

SV	ME	MSE	NMSE [%]
EA	0.040	**0.045**	**3.115**
SFLC	**0.000**	0.060	4.300
AF	0.250	1.065	77.285
SNN	−0.305	0.135	8.965
GRU NN	−0.165	0.085	5.645

SV deviation calculated to trend line for the signal processing methods of the *ergometry test*; best values are marked in bold, SV values are normalized.

## Data Availability

The data presented in this study are available on request from the corresponding author.

## Supplementary Materials

The following are available online at https://www.mdpi.com/article/10.3390/s22207883/s1, Figure S1. Increase of blood vessel diameter due to pulse wave within the descending aorta, Figure S2. Orientation of red blood cells during diastole and flow of electrical current, Figure S3. Orientation of red blood cells during systole and flow of electrical current, Figure S4. Electrocardiogram (ECG) and bioimpedance signal—fiducial points of the ECG (R-wave of the QRS-complex), and the impedance cardiography (ICG) signal (B-, C-, and X-point) are marked, with B-point to X-point representing the left ventricular ejection time (LVET), and the C-point the maximum of the acceleration and change of impedance of the blood, Figure S5. Example simulation of surface potential (left, 4 different visualizations, first dB scaling, second linear scaling, third and fourth with equipotential lines) and current density (right) from the human model simulated with Sim4Life and the human phantom DUKE model (Computable Virtual Population V3.x—cViP 3.x models) provided by the Foundation for Research on Information Technologies in Society (IT‘IS).

## Abbreviations

The following abbreviations are used in this manuscript:

AF	adaptive filter
CCM	cardiometry current meter
CT	computer tomography
CVD	cardiovascular disease
EA	ensemble averaging
ECG	electrocardiogram
EMD	empirical mode decomposition
GRU	gated recurrent unit
ICA	independent component analysis
ICG	impedance cardiography
ICU	intensive care unit
LMS	least mean square
LSTM	long-short term memory
ME	mean error
MRI	magnetic resonance imaging
MSE	mean square error
NCD	non-communicable disease
NMSE	normalized mean square error
NN	neural network
PCA	principal component analysis
PPG	photoplethysmogram
ReLU	rectified linear unit
RMS	root mean square
RNN	recurrent neural network
SFLC	scaled Fourier linear combiner
SNN	shallow neural network
SNR	signal to noise ratio
STD	standard deviation
SV	stroke volume
TEB	thoracic electrical bioimpedance
