# Supplementary material for: GRU Neural Network Improved Bioimpedance Based Stroke Volume Estimation during Ergometry Stress Test
Source: Sensors (Basel). 2022 Oct 17;22(20):7883. doi: 10.3390/s22207883 (PMC9612153; doi:10.3390/s22207883)
Supplement: Supplementary file 1 [file sensors-22-07883-s001.zip › sensors-1951895-supplementary.pdf]

ICG Fundamentals

The bioimpedance signal contains different components. A baseline resistance due to the general tissue, an alternating resistive part due to respiration, and an alternating resistive part due to the cardiac activity [1] (p. 135). First, it is assumed that the change of the bioimpedance due to the cardiac activity is related to the change in the blood vessel diameter [2]. For the thoracic electrical bioimpedance (TEB), this is the blood volume change in the descending aorta (see **Figure S1**). Blood is a relatively good electrical conductor compared to the surrounding tissue with 0.7 S/m [3] (p. 88), and so the measured body resistance will drop along the descending aorta because of the increase of blood volume during the systolic phase, leading to an increase of conductivity in this area.

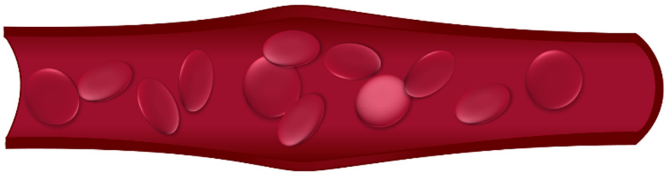

Figure S1. Increase of blood vessel diameter due to pulse wave within the descending aorta.

Beyond the volume change, a second effect occurs in the aorta that impacts the blood's conductivity. The orientation of the erythrocytes is due to the blood acceleration and shear forces during the opening of the aortic valve in the systolic phase [4] (see **Figure S2** and **Figure S3**). Blood behaves like an anisotropic conductor during the systolic phase and increases its conductivity along the descending aorta depending on the blood acceleration due to applied pressure. In general, many tissues behave like an anisotropic conductor due to the tissue structure, cell shape, and lipid membrane surface.

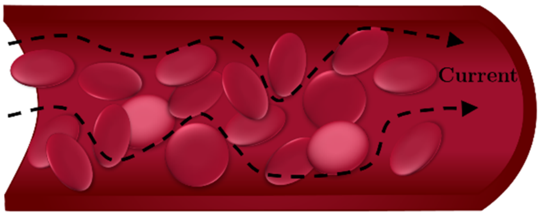

Figure S2. Orientation of red blood cells during diastole and flow of electrical current, inspired by [1] (p. 141).

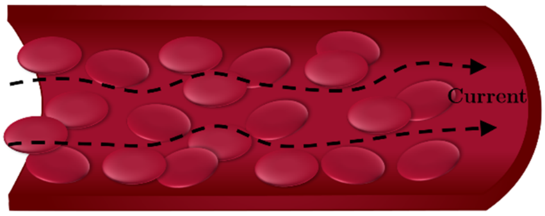

Figure S3. Orientation of red blood cells during systole and flow of electrical current, inspired by [1] (p. 141).

ICG delivers a signal, and its fiducial points are used to estimate hemodynamic management parameters as stroke volume (SV) of a human subject. According to Bernstein and Osypka in [1] (p. 143), SV is calculated by

$$SV = V_{EPT} \cdot \sqrt{\frac{\left|\left(\frac{dZ(t)}{dt}\right)_{MIN}\right|}{Z_0}} \cdot FT_C, \qquad \text{(S1)}$$

where V_{EPT} is the volume of electrically participating tissue, $\left(\frac{\mathrm{dZ(t)}}{\mathrm{dt}}\right)_{\mathrm{MIN}}$ is the maximal amplitude (C-point) of the derivative of the high pass filtered and inverted bioimpedance signal, Z_0 is the baseline impedance, and FT_C is the corrected flow time, this is the left ventricular ejection time (time difference between B- and X-point) divided by the square root of the R-R-interval, see **Figure S4**. The index of $\frac{\mathrm{dZ(t)}}{\mathrm{dt}}$ is not standardized in the literature. Bernstein and Osypka in [1] (p. 143) use 'min' to indicate that the derivative impedance has its lowest value, a negative one. Due to the signal $\frac{\mathrm{dZ(t)}}{\mathrm{dt}}$ being normally inverted but sometimes not mentioned, one can often see the index 'max' in the literature.

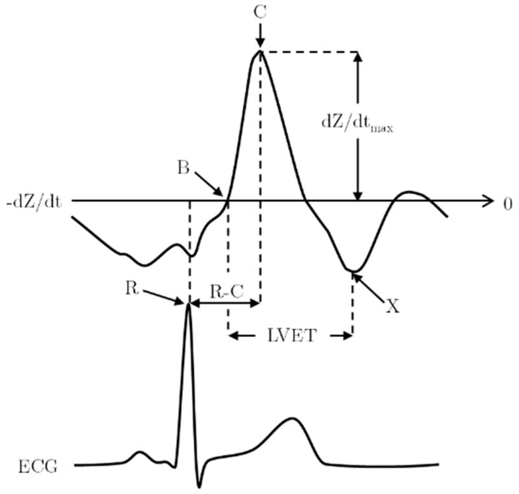

Figure S4. Electrocardiogram (ECG) and bioimpedance signal – fiducial points of the ECG (R-wave of the QRS-complex), and the impedance cardiography (ICG) signal (B-, C-, and X-point) are marked, with B-point to X-point representing the left ventricular ejection time (LVET), and the C-point the maximum of the acceleration and change of impedance of the blood.

Different other methods exist to calculate the stroke volume, e.g., thermodilution (as the gold standard), pulse contour analysis, esophageal Doppler, and the Fick method [5].

These methods have different disadvantages as some are invasive, expensive and many of them are not capable of estimating the SV beat-to-beat. The bioimpedance measurement, in contrast, has its own limitations as it is sensitive to movement artifacts and can suffer from incorrect and suboptimal electrode placement. We have investigated the current distribution [6] and measurement robustness with displaced electrodes [7] in previous work. This was done to find an optimal position for the electrode setup that is robust in terms of electrode displacement and movement artifacts, results in a large amount of current passing the descending aorta, and is simply applicable in clinical practice (see **Figure S5**, electrodes are named A, B, C, and D).

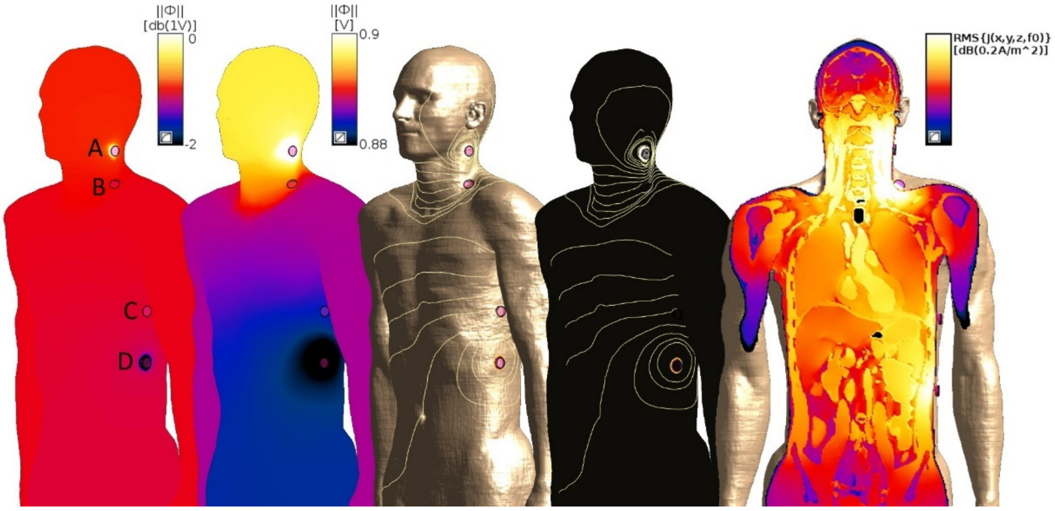

Figure S5. Example simulation of surface potential (left, 4 different visualizations, first dB scaling, second linear scaling, third and fourth with equipotential lines) and current density (right) from the human model simulated with Sim4Life [8] and the human phantom DUKE model (Computable Virtual Population V3.x – cViP 3.x models) provided by the Foundation for Research on Information Technologies in Society (IT'IS) [9] [10].

We also evaluated the possible estimation of the bioimpedance signal during disturbances with a long-short term memory (LSTM) network [11] during ergometry. This was done with a multi-channel voltage measurement. With this setup, we evaluated the feasibility of the LSTM approach for a single subject within a laboratory environment during an exercise stress test. This is the groundwork for the current topic. We want to estimate the bioimpedance signal and SV under adverse conditions as in the *ergometry stress test* that results in a large number of heavy artifacts in the signal with multiple human subjects. Compared to the former work, we used 23 human subjects to investigate inter-subject phenomena, test algorithm robustness, and improve the evaluation step. Whereas in the former work, only the undisturbed intended breaks during the stress test were evaluated, we will now use interpolation to check the quality of the estimated and reconstructed signal between the breaks.

Additionally, a different test protocol was created to investigate the estimated bioimpedance signals after processing with a clear reference. This test was performed with the later used stress test setup, and the electrodes of the subject were affected by push and pull forces that resulted in a change of the skin-electrode interface and changes in the point of measurement. The point of measurement (measurement point of surface potential) is important if we consider that a small change in this point will result in a large difference in measured bioimpedance. As an example, the baseline impedance between electrodes B and C can be estimated as 30 Ω; the distance is assumed to be 20 cm for the example. This results in 1.5 Ω per cm. If we keep in mind that the bioimpedance signal dZ has an amplitude of, e.g., 0.2 Ω, a shift of the point of measurement of 1.33 mm results in an artifact as large as the bioimpedance signal. With a glued electrode, a small force on the electrode will shift the electrode gel distribution and may also stretch the skin minimally. These small changes can cause an artifact. The closer the voltage measurement electrodes B, and C are to the current injecting electrodes A and B; the more susceptible the system is to artifacts due to the compressed equipotential lines on the surface, shown in the example **Figure S5**. The push and pull force disturbances seem to have a very large contribution to artifacts in bioimpedance signals. Other sources of error can be a very limited output resistance of the current source, the capacitive coupling between patient leads, the demodulation method, and, e.g., electromagnetic interference (EMI) effects.

Abbreviations

The following abbreviations are used in this manuscript:

ECG	electrocardiogram
EMI	electromagnetic interference
GRU	gated recurrent unit
ICG	impedance cardiography
LSTM	long-short term memory
LVET	left ventricular ejection time
SV	stroke volume
TEB	thoracic electrical bioimpedance

References

1. Kleinman, C. & Seri, I. Hemodynamics and Cardiology: Neonatology Questions and Controversies 2nd Edition Saunders, 2012
2. Katsuyuki Sakamoto and Hiroshi Kanai. “Electrical Characteristics of Flowing Blood.” In: IEEE Transactions on Biomedical Engineering BME-26.12 (1979), pp. 686–695. issn: 0018-9294. doi: 10.1109/tbme. 1979.326459.
3. Sverre Grimnes and Ørjan Grøttem Martinsen. Bioimpedance and Bioelectricity Basics 3rd ed. Academic Press, 2015. url: http://cds. cern.ch/record/1970152.
4. Bernstein, D. P. Impedance cardiography: Pulsatile blood flow and the biophysical and electrodynamic basis for the stroke volume equations Journal of Electrical Bioimpedance, 2010, 1, 2-17
5. Garcia, X.; Mateu, L.; Maynar, J.; Mercadal, J.; Ochagavia, A. & Ferrandiz, A. Estimating cardiac output. Utility in the clinical practice. Available invasive and non-invasive monitoring, Medicina Intensiva, Elsevier BV, 2011, 35, 552-561
6. Urban, M. & Orglmeister, R. Evaluation of Electrode Setups by MRI Based Human Phantom with FEM Based Quasi-Static Solver for Bioimpedance Measurement 2019 41st Annual International Conference of the IEEE Engineering in Medicine and Biology Society (EMBC), IEEE, 2019
7. Urban, M. & Orglmeister, R. Surface Potential Simulation for Robust Electrode Placement by MRI Based Human Phantom with FEM Based Quasi-Static Solver for Bioimpedance Measurement 2019 41st Annual International Conference of the IEEE Engineering in Medicine and Biology Society (EMBC), IEEE, 2019
8. Christ, Andreas, et al. "The Virtual Family - development of surface-based anatomical models of two adults and two children for dosi¬metric simulations."Physics in Medicine & Biology 55.2 (2009): N23.
9. Gosselin, M.-C., et al., Development of a new generation of high-re¬solution anatomical models for medical device evaluation: the Virtual Population 3.0., Physics in Medicine & Biology 59.18 (2014): 5287.
10. https://zmt.swiss/sim4life/ (accessed 2018-11-14)
11. Urban, M.; Tigges, T.; Klum, M.; Pielmus, A. & Orglmeister, R. Improvement of Stroke Volume Estimation with Bioimpedance Measurement by LSTM Network Approach Based on ECG during Ergometry Current Directions in Biomedical Engineering, Walter de Gruyter GmbH, 2019, 5, 33-36
